# Supplementary material for: Mesenchymal–Epithelial Transition Kinase Inhibitor Therapy in Patients with Advanced Papillary Renal-Cell Carcinoma: A Systematic Review and Meta-Analysis
Source: Int J Mol Sci. 2023 Dec 18;24(24):17582. doi: 10.3390/ijms242417582 (PMC10744118; doi:10.3390/ijms242417582)
Supplement: Supplementary file 1 [file ijms-24-17582-s001.zip › ijms-2676607-supplementary.pdf]

Supplementary Material

Table S1: Search strategies

Table S2. Critical appraisal of RCTs according to the Cochrane Collaboration's tool for assessing risk of bias in RCTs (Rob2).

Table S3. Critical appraisal of non-RCTs according to the Cochrane Collaboration's tool for assessing risk of bias in non-RCTs (ROBINS-I).

Figure S1: leave-one-out sensitive-analysis of ORR

Figure S2: leave-one-out sensitive-analysis of DCR

Figure S3: leave-one-out sensitive-analysis of PFS 12 in months

Figure S4: leave-one-out sensitive-analysis of OS 12 in months

Figure S5: leave-one-out sensitive-analysis of OS 24 in months

Figure S6: funnel plot of ORR

Figure S7: funnel plot of DCR

Figure S8: funnel plot of PFS

Figure S9: funnel plot of OS 12 months

Figure S10: funnel plot of OS 24 months

Figure S11. Any grade of adverse events. **A.** Vomiting **B.** Rash **C.** Proteinuria **D.** Nausea **E.** Hyponatremia. **F.** Fatigue. **G.** Edema. **H.** Diarrhea. **I.** Diarrhea. **J.** Increased AST. **K.** Anorexia**.** **L.** Anemia. **M.** Increased ALT.

Figure S12. Grade ≥3 adverse events. **A.** Decreased WBC. **B.** Vomiting. **C.** Rash. **D.** Proteinuria. **E.** Decreased neutrophil Count. **F.** Nausea. **G.** Mucositis. **H.** Hyponatremia. **I.** Hypoalbuminemia. **J.** Hypertension. **K.** Fatigue. **L.** Extremity pain. **M.** Edema **N.** Dyspnea. **O.** Diarrhea. **P.** Increased creatinine. **Q.** Increased AST. **R.** Anorexia. **S.** Anemia. **T.** Increased ALT. **U.** Abdominal pain.

Table S1: Search strategies.

Database	Search Strategy
**PubMed**	(((((((((((((((((((((((((((("Papillary renal cell carcinoma"[MeSH Terms]) OR ("Papillary renal cell carcinoma"[Title/Abstract])) OR ("Papillary renal cell carcinoma, multiple"[Title/Abstract])) OR ("Papillary renal cell carcinoma, familial"[Title/Abstract])) OR ("Papillary renal carcinoma, malignant"[Title/Abstract])) OR ("Papillary renal cell carcinoma, bilateral"[Title/Abstract])) OR ("Carcinoma, Renal Cell"[MeSH Terms])) OR ("Carcinoma, Renal Cell"[Title/Abstract])) OR ("Carcinomas, Renal Cell"[Title/Abstract])) OR ("Renal Cell Carcinomas"[Title/Abstract])) OR ("Renal Carcinomas"[Title/Abstract])) OR ("Kidney Neoplasms"[MeSH Terms])) OR ("Kidney Neoplasms"[Title/Abstract])) OR ("Kidney Neoplasm"[Title/Abstract])) OR ("Neoplasm, Kidney"[Title/Abstract])) OR ("Renal Neoplasms"[Title/Abstract])) OR ("Neoplasm, Renal"[Title/Abstract])) OR ("Neoplasms, Renal"[Title/Abstract])) OR ("Renal Neoplasm"[Title/Abstract])) OR ("Neoplasms, Kidney"[Title/Abstract])) OR ("Cancer of Kidney"[Title/Abstract])) OR ("Kidney Cancers"[Title/Abstract])) OR ("Renal Cancer"[Title/Abstract])) OR ("Cancer, Renal"[Title/Abstract])) OR ("Cancers, Renal"[Title/Abstract])) OR ("Renal Cancers"[Title/Abstract])) OR ("Cancer of the Kidney"[Title/Abstract])) OR ("Kidney Cancer"[Title/Abstract])) OR ("Cancer, Kidney"[Title/bstract])) OR ("Cancers, Kidney"[Title/Abstract])) AND (((((((((((((((((("c-MET inhibitor"[Title/Abstract])) OR (("c-MET inhibitors"[Title/Abstract]))) OR (("MET inhibitor"[Title/Abstract]))) OR (("MET inhibitors"[Title/Abstract]))) OR (("Cabozantinib"[Title/Abstract]))) OR (("Crizotinib"[Title/Abstract]))) OR (("Crizotinib")[MeSH Terms])) OR (("Cabozantinib")[MeSH Terms]))) OR (("Tivantinib"[Title/Abstract]))) OR (("Foretinib"[Title/Abstract]))) OR (("Savolitinib"[Title/Abstract]))) OR (("AMG-208")[MeSH Terms])) OR (("AMG-208"[Title/Abstract]))) OR (("PF-04217903"[Title/Abstract]))) OR (("BMS777607"[Title/Abstract]))) OR (("NVP-BVU972"[Title/Abstract]))) OR (("PHA-665752"[Title/Abstract]) OR ("MET-directed agents" [Title/Abstract]))
**Scopus**	(("Papillary renal cell carcinoma") OR TITLE-ABS-KEY("Papillary renal cell carcinoma") OR TITLE-ABS-KEY("Papillary renal cell carcinoma, multiple") OR TITLE-ABS-KEY("Papillary renal cell carcinoma, familial") OR TITLE-ABS-KEY("Papillary renal cell carcinoma, malignant") OR TITLE-ABS-KEY("Papillary renal cell carcinoma, bilateral") OR MESH("Carcinoma, Renal Cell") OR TITLE-ABS-KEY("Carcinoma, Renal Cell") OR TITLE-ABS-KEY("Carcinomas, Renal Cell") OR TITLE-ABS-KEY("Renal Cell Carcinomas") OR TITLE-ABS-KEY("Renal Carcinomas") OR MESH("Kidney Neoplasms") OR TITLE-ABS-KEY("Kidney Neoplasms") OR TITLE-ABS-KEY("Kidney Neoplasm") OR TITLE-ABS-KEY("Neoplasm, Kidney") OR TITLE-ABS-KEY("Renal Neoplasms") OR TITLE-ABS-KEY("Neoplasm, Renal") OR TITLE-ABS-KEY("Neoplasms, Renal") OR TITLE-ABS-KEY("Renal Neoplasm") OR TITLE-ABS-KEY("Neoplasms, Kidney") OR TITLE-ABS-KEY("Cancer of Kidney") OR TITLE-ABS-KEY("Kidney Cancers") OR TITLE-ABS-KEY("Renal Cancer") OR TITLE-ABS-KEY("Cancer, Renal") OR TITLE-ABS-KEY("Cancers, Renal") OR TITLE-ABS-KEY("Renal Cancers") OR TITLE-ABS-KEY("Cancer of the Kidney") OR TITLE-ABS-KEY("Kidney Cancer") OR

	TITLE-ABS-KEY("Cancer, Kidney") OR TITLE-ABS-KEY("Cancers, Kidney"))) AND ((TITLE-ABS-KEY("c-MET inhibitor") OR TITLE-ABS-KEY("c-MET inhibitors") OR TITLE-ABS-KEY("MET inhibitor") OR TITLE-ABS-KEY("MET inhibitors") OR TITLE-ABS-KEY("Cabozantinib") OR TITLE-ABS-KEY("Crizotinib") OR TITLE-ABS-KEY("AMG-208") OR TITLE-ABS-KEY("PF-04217903") OR TITLE-ABS-KEY("BMS777607") OR TITLE-ABS-KEY("NVP-BVU972") OR TITLE-ABS-KEY("PHA-665752") OR TITLE-ABS-KEY("MET-directed agents")))
**The Cochrane Library**	("Papillary renal cell carcinoma"):ti,ab,kw OR ("Papillary renal cell carcinoma"):ti,ab,kw OR ("Papillary renal cell carcinoma, multiple"):ti,ab,kw OR ("Papillary renal cell carcinoma, familial"):ti,ab,kw OR ("Papillary renal cell carcinoma, malignant"):ti,ab,kw OR ("Papillary renal cell carcinoma, bilateral"):ti,ab,kw OR ("Carcinoma, Renal Cell"):ti,ab,kw OR ("Carcinoma, Renal Cell"):ti,ab,kw OR ("Carcinomas, Renal Cell"):ti,ab,kw OR ("Renal Cell Carcinomas"):ti,ab,kw OR ("Renal Carcinomas"):ti,ab,kw OR ("Kidney Neoplasms"):ti,ab,kw OR ("Kidney Neoplasms"):ti,ab,kw OR ("Kidney Neoplasm"):ti,ab,kw OR ("Neoplasm, Kidney"):ti,ab,kw OR ("Renal Neoplasms"):ti,ab,kw OR ("Neoplasm, Renal"):ti,ab,kw OR ("Neoplasms, Renal"):ti,ab,kw OR ("Renal Neoplasm"):ti,ab,kw OR ("Neoplasms, Kidney"):ti,ab,kw OR ("Cancer of Kidney"):ti,ab,kw OR ("Kidney Cancers"):ti,ab,kw OR ("Renal Cancer"):ti,ab,kw OR ("Cancer, Renal"):ti,ab,kw OR ("Cancers, Renal"):ti,ab,kw OR ("Renal Cancers"):ti,ab,kw OR ("Cancer of the Kidney"):ti,ab,kw OR ("Kidney Cancer"):ti,ab,kw OR ("Cancer, Kidney"):ti,ab,kw OR ("Cancers, Kidney"):ti,ab,kw) AND ("c-MET inhibitor"):ti,ab,kw OR ("c-MET inhibitors"):ti,ab,kw OR ("MET inhibitor"):ti,ab,kw OR ("MET inhibitors"):ti,ab,kw OR ("Cabozantinib"):ti,ab,kw OR ("Crizotinib"):ti,ab,kw OR ("AMG-208"):ti,ab,kw OR ("PF-04217903"):ti,ab,kw OR ("BMS777607"):ti,ab,kw OR ("NVP-BVU972"):ti,ab,kw OR ("PHA-665752"):ti,ab,kw OR ("MET-directed agents"):ti,ab,kw)

Table S2: Critical appraisal of RCTs according to the Cochrane Collaboration's tool for assessing risk of bias in RCTs (Rob2). Datas were sourced from reference [1–3].

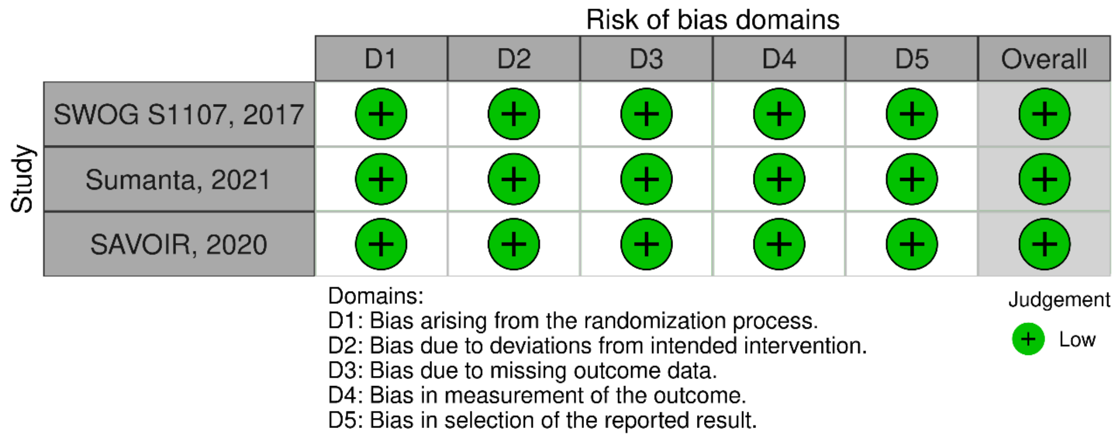

Table S3: Critical appraisal of non-RCTs according to the Cochrane Collaboration's tool for assessing risk of bias in non-RCTs (ROBINS-I). Datas were sourced from reference [4–9]

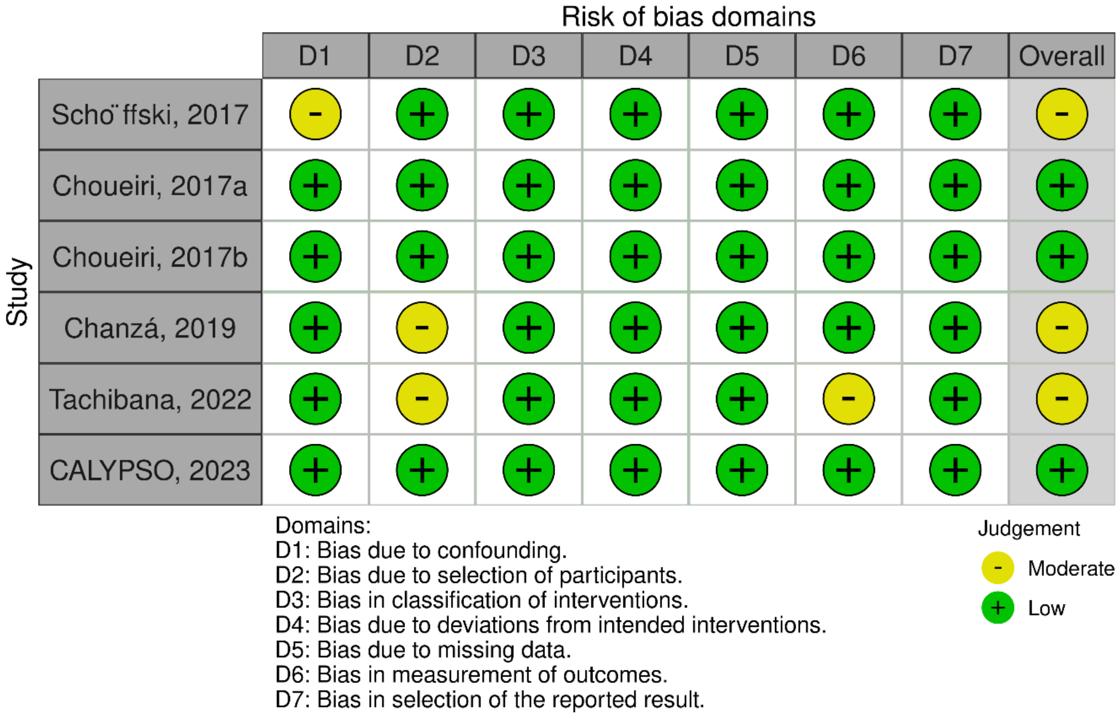

Figure S1: leave-one-out sensitive-analysis of ORR. Datas were sourced from reference [1,2,4–10]

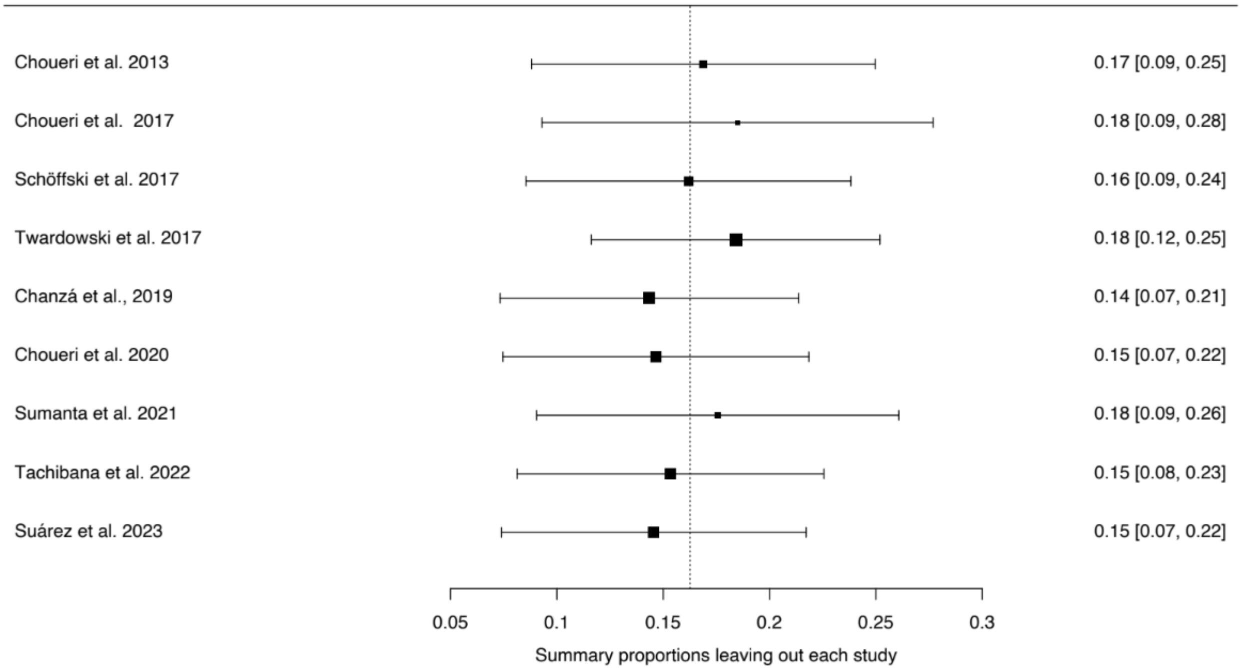

Figure S2: leave-one-out sensitive-analysis of DCR. Datas were sourced from reference [2,4–9]

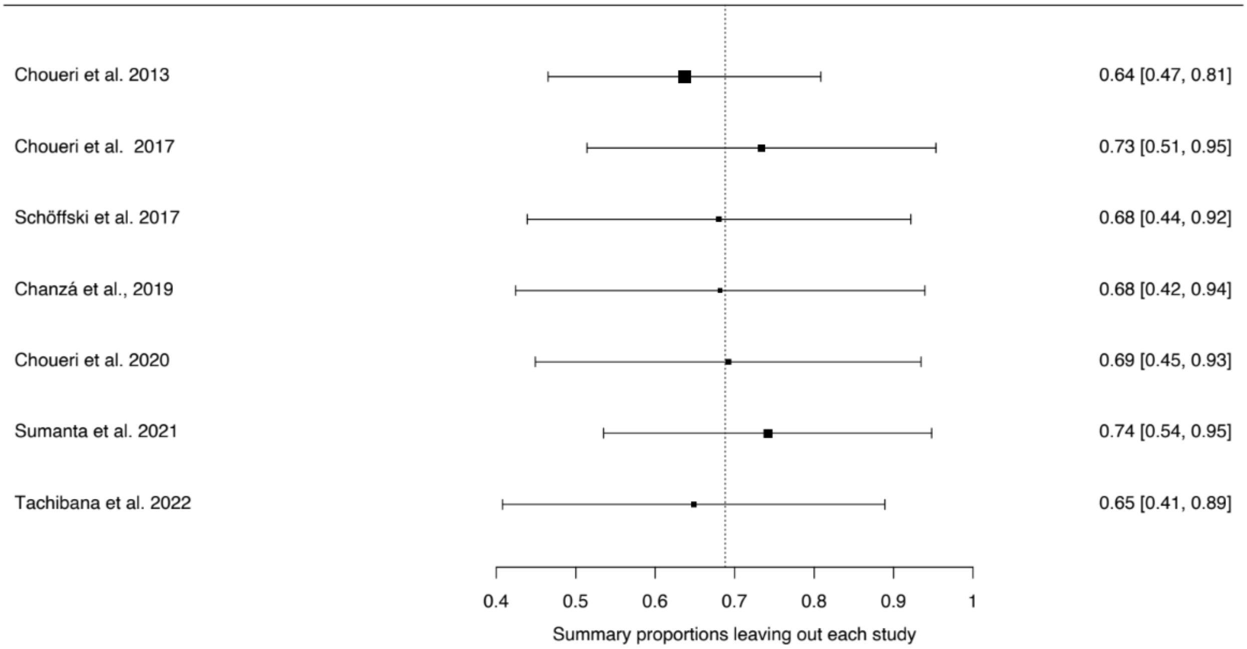

Figure S3: leave-one-out sensitive-analysis of PFS. Datas were sourced from reference [2,4–9].

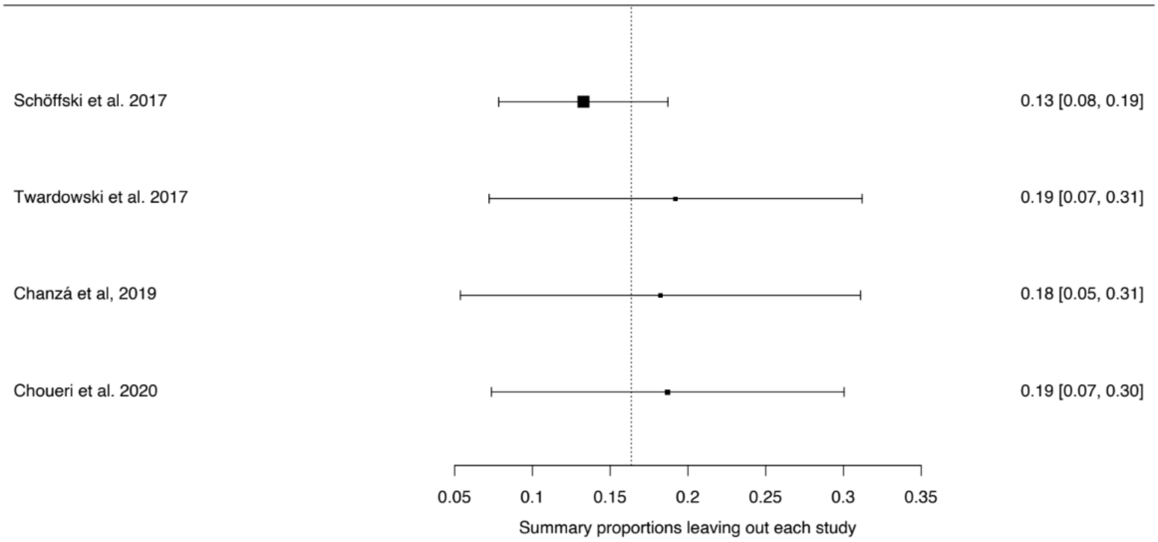

Figure S4: leave-one-out sensitive-analysis of OS 12 months. Datas were sourced from reference [1,4,7,9].

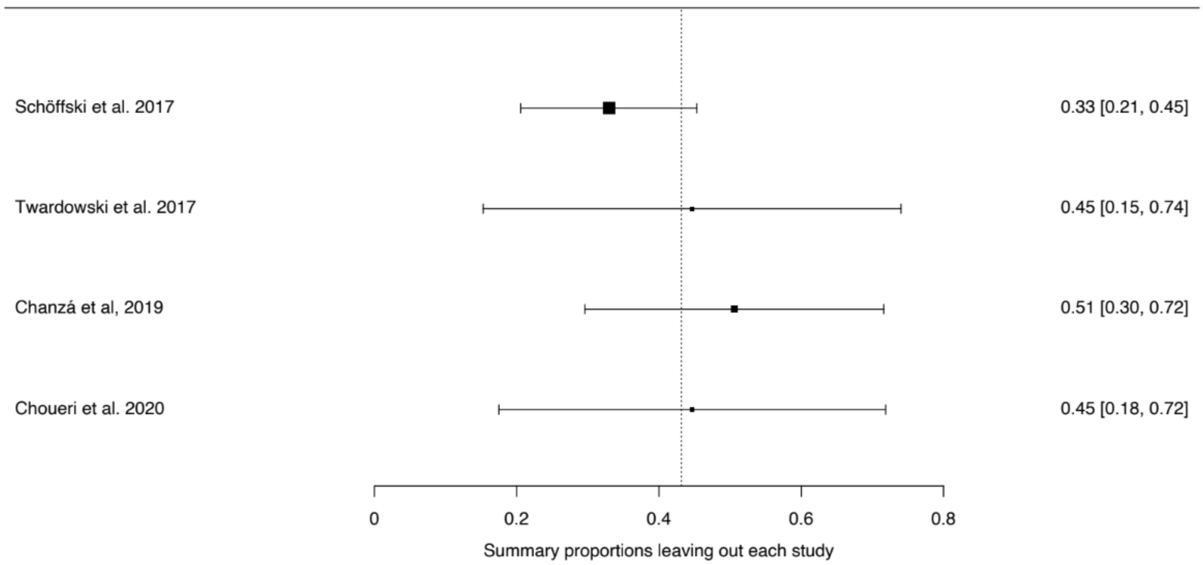

Figure S5: leave-one-out sensitive-analysis of OS 24 months. Datas were sourced from reference [4,7,9].

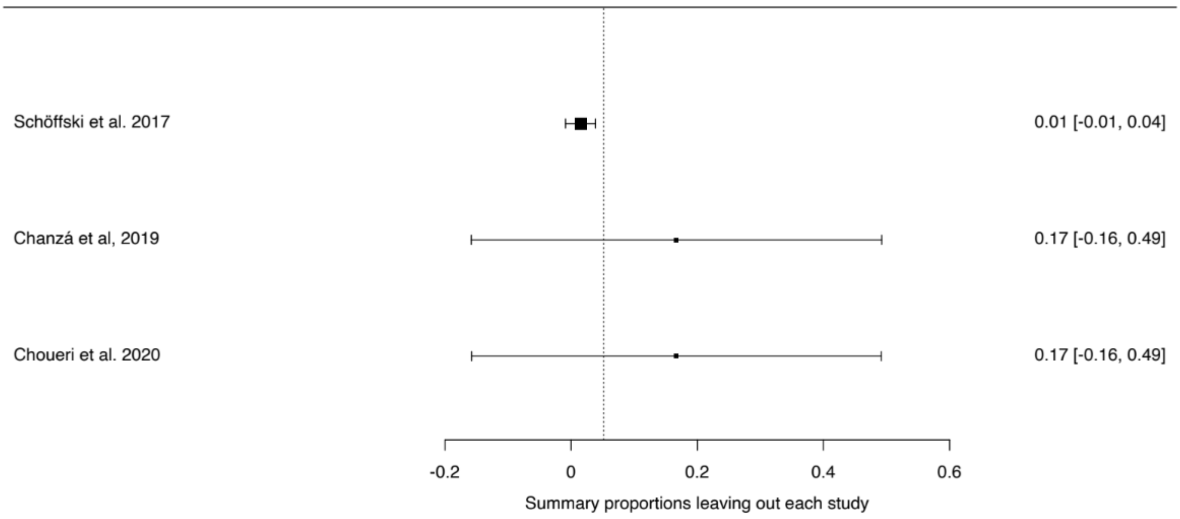

Figure S6: funnel plot of ORR. Datas were sourced from reference [1,6,7].

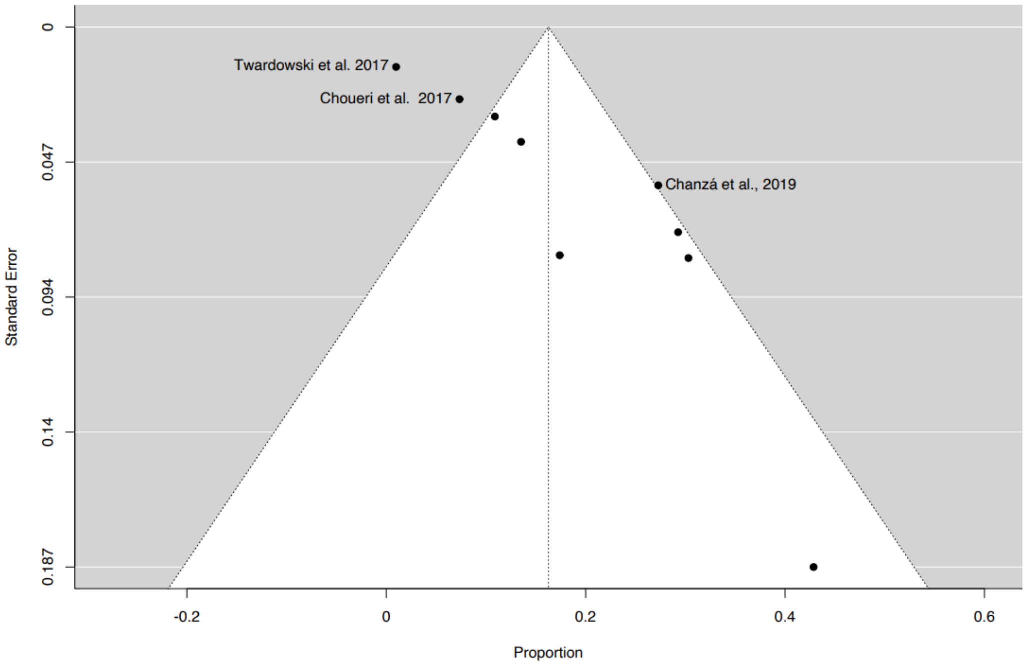

Figure S7: funnel plot of DCR. Datas were sourced from reference [2,6,8,9].

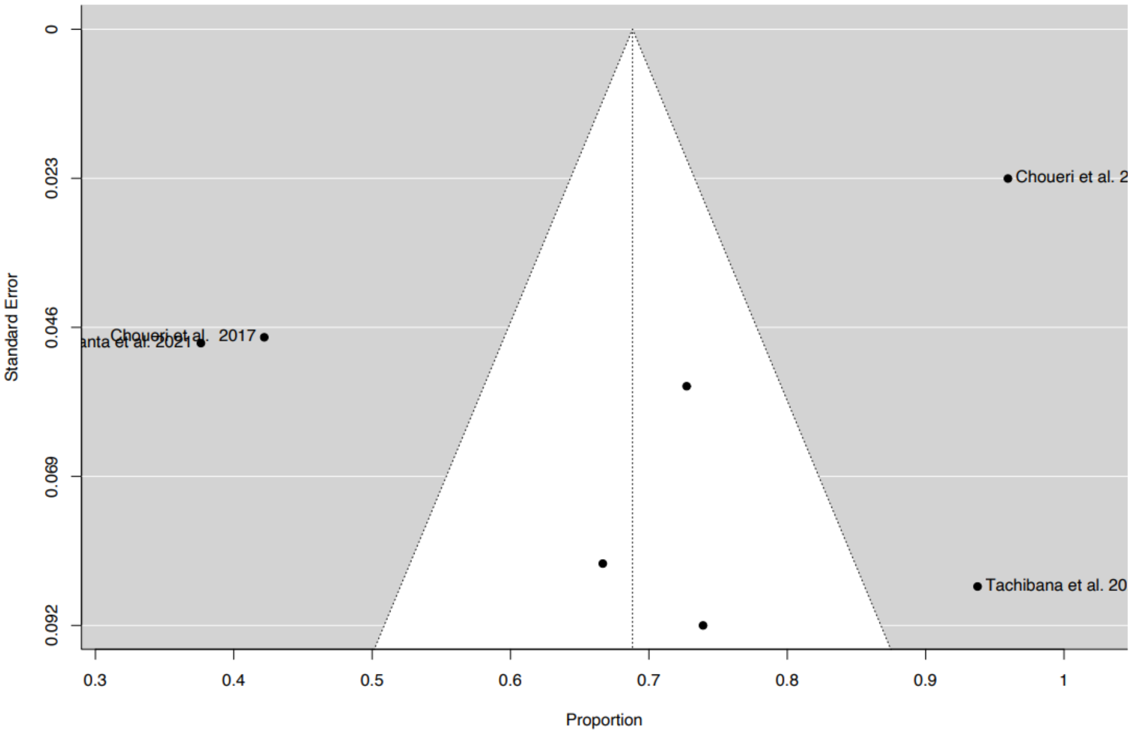

Figure S8: Funnel plot of PFS. Datas were sourced from reference [4].

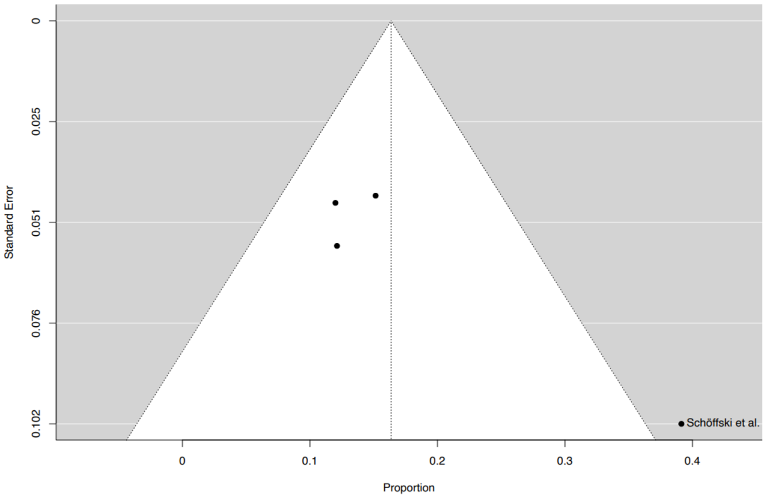

Figure S9: Funnel plot of OS 12 months. Datas were sourced from reference [4,7].

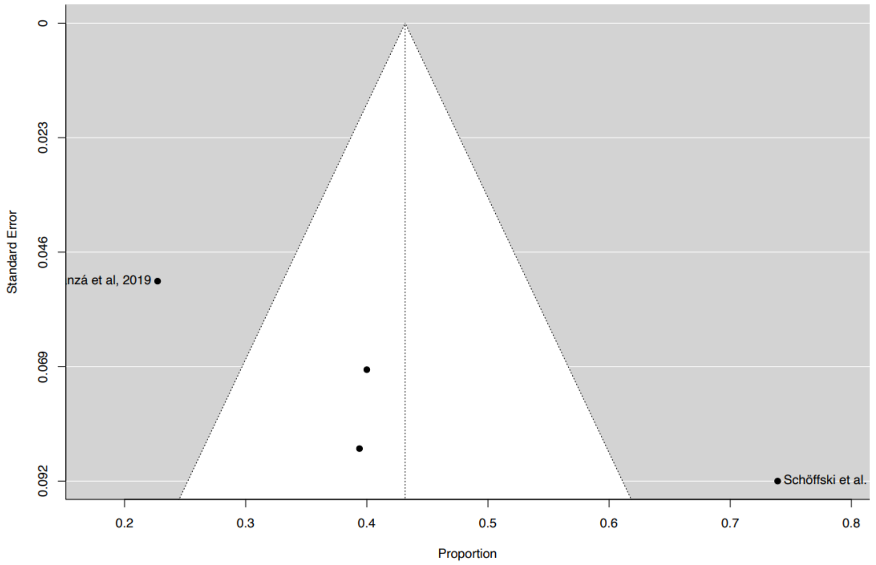

Figure S10: Funnel plot of OS 24 months. Datas were sourced from reference [4,7].

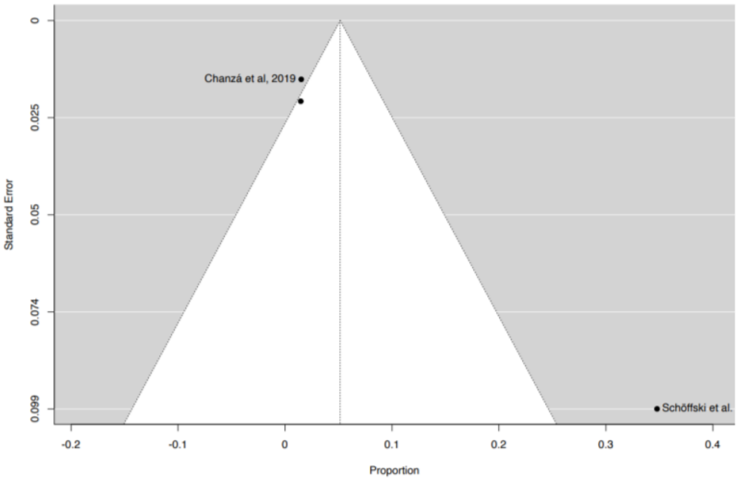

Figure S11. Any grade of adverse events. **A.** Vomiting **B.** Rash **C.** Proteinuria **D.** Nausea **E.** Hyponatremia. **F.** Fatigue. **G.** Edema. **H.** Diarrhea. **I.** Diarrhea. **J.** Increased AST. **K.** Anorexia. **L.** Anemia. **M.** Increased ALT. Datas were sourced from reference [2,4–6].

A. Vomiting

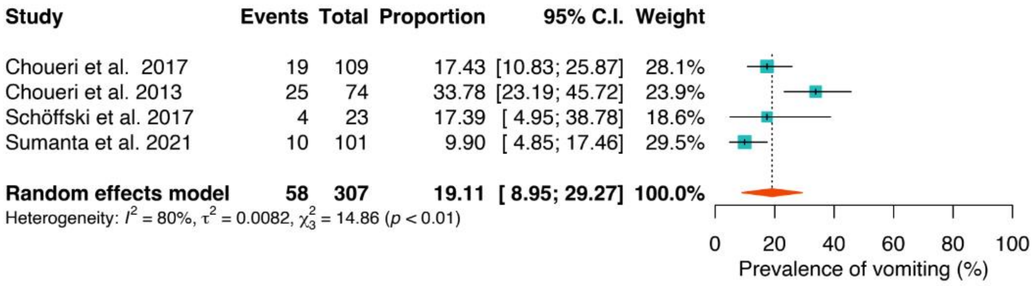

B. Rash

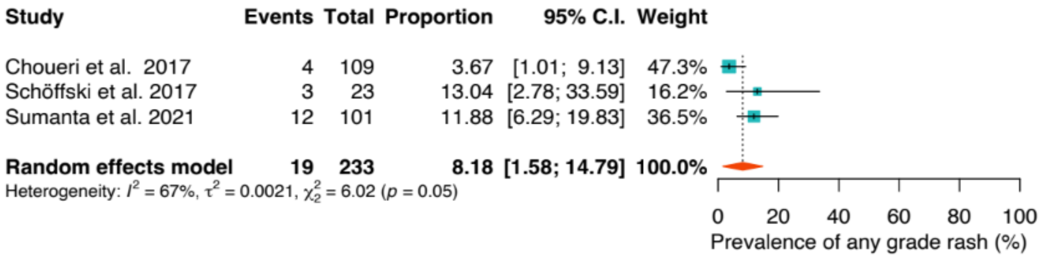

C. Proteinuria

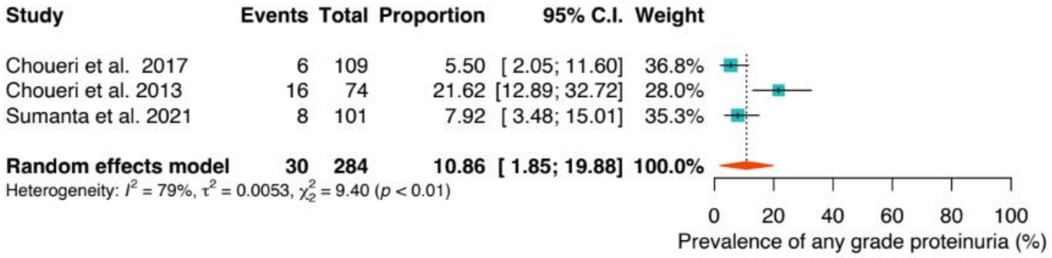

D. Nausea

Study	Events	Total	Proportion	95% C.I.	Weight
Choueri et al. 2017	42	109	38.53	[29.37; 48.34]	30.8%
Choueri et al. 2013	38	74	51.35	[39.44; 63.15]	25.8%
Schöffski et al. 2017	11	23	47.83	[26.82; 69.41]	12.7%
Sumanta et al. 2021	34	101	33.66	[24.56; 43.75]	30.7%
**Random effects model**	**125**	**307**	**41.53**	**[33.02; 50.03]**	**100.0%**

Heterogeneity: $I^2 = 52\%$, $\tau^2 = 0.0039$, $\chi^2_3 = 6.27$ ($p = 0.10$)

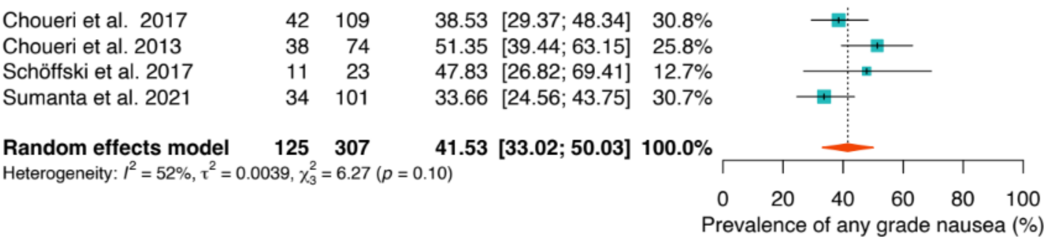

E. Hyponatremia

Study	Events	Total	Proportion	95% C.I.	Weight
Choueri et al. 2017	5	109	4.59	[1.51; 10.38]	37.2%
Schöffski et al. 2017	9	23	39.13	[19.71; 61.46]	26.4%
Sumanta et al. 2021	10	101	9.90	[4.85; 17.46]	36.4%
**Random effects model**	**24**	**233**	**15.64**	**[0.00; 34.28]**	**100.0%**

Heterogeneity: $I^2 = 84\%$, $\tau^2 = 0.0239$, $\chi^2_2 = 12.37$ ($p < 0.01$)

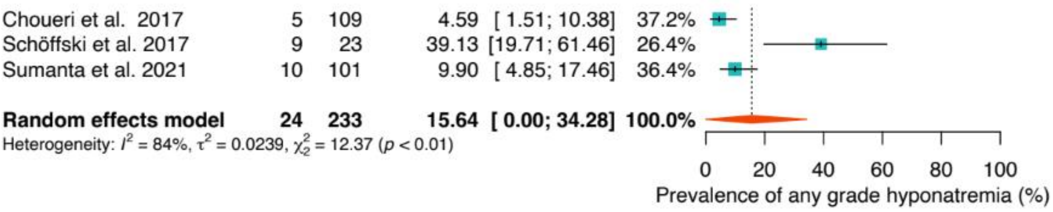

F. Fatigue

Study	Events	Total	Proportion	95% C.I.	Weight
Choueri et al. 2017	23	109	21.10	[13.87; 29.96]	26.3%
Choueri et al. 2013	54	74	72.97	[61.39; 82.65]	25.7%
Schöffski et al. 2017	12	23	52.17	[30.59; 73.18]	22.1%
Sumanta et al. 2021	57	101	56.44	[46.20; 66.28]	25.8%
**Random effects model**	**146**	**307**	**50.45**	**[28.27; 72.62]**	**100.0%**

Heterogeneity: $I^2 = 96\%$, $\tau^2 = 0.0471$, $\chi^2_3 = 72.60$ ($p < 0.01$)

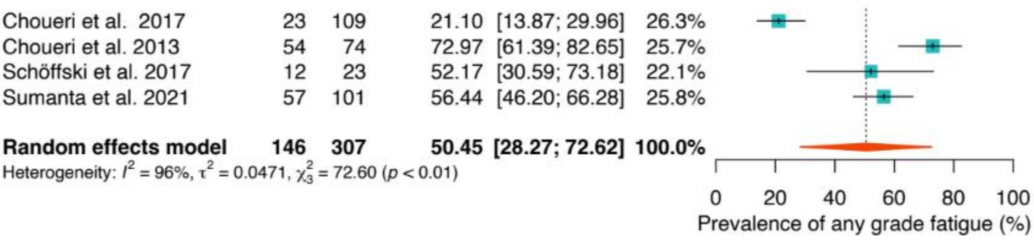

G. Edema

Study	Events	Total	Proportion	95% C.I.	Weight
Choueri et al. 2017	3	109	2.75	[0.57; 7.83]	36.0%
Schöffski et al. 2017	11	23	47.83	[26.82; 69.41]	29.0%
Sumanta et al. 2021	17	101	16.83	[10.12; 25.58]	35.1%
**Random effects model**	**31**	**233**	**20.76**	**[0.00; 45.45]**	**100.0%**

Heterogeneity: $I^2 = 93\%$, $\tau^2 = 0.0439$, $\chi^2_2 = 28.84$ ($p < 0.01$)

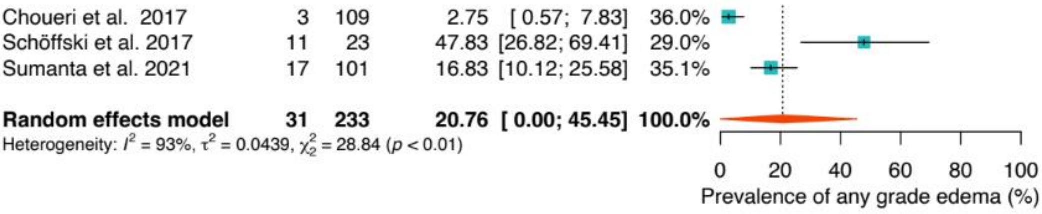

H. Diarrhea

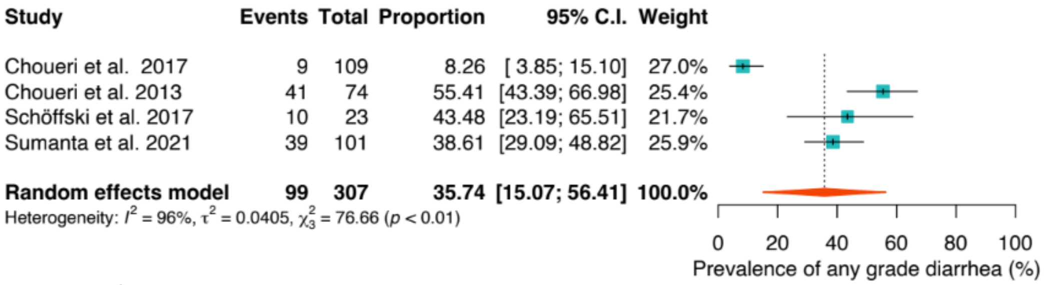

I. Increased creatinine

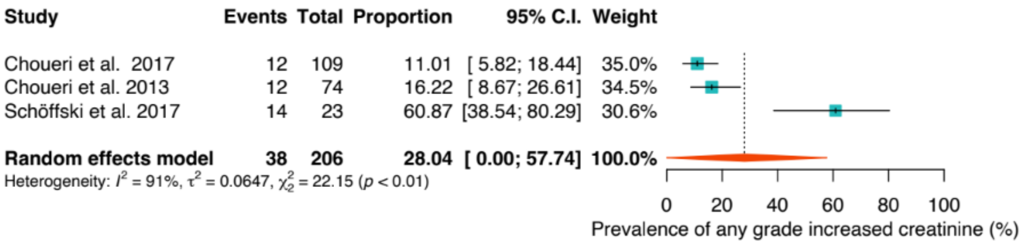

J. Increased AST

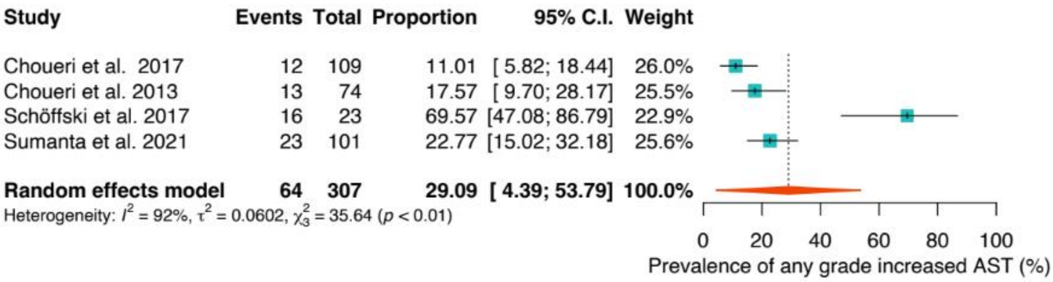

K. Anorexia

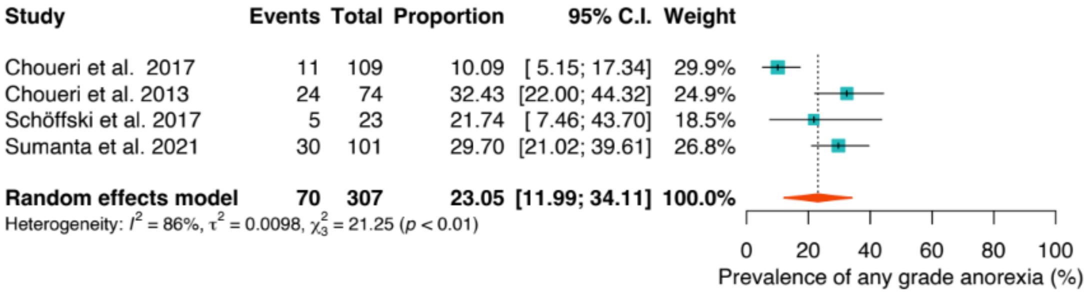

L. Anemia

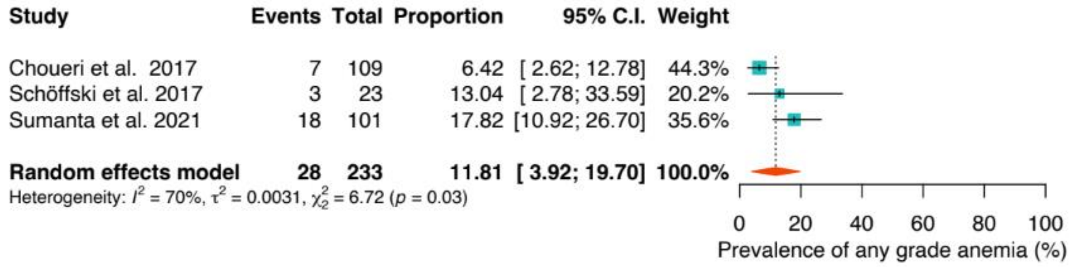

M. Increased ALT

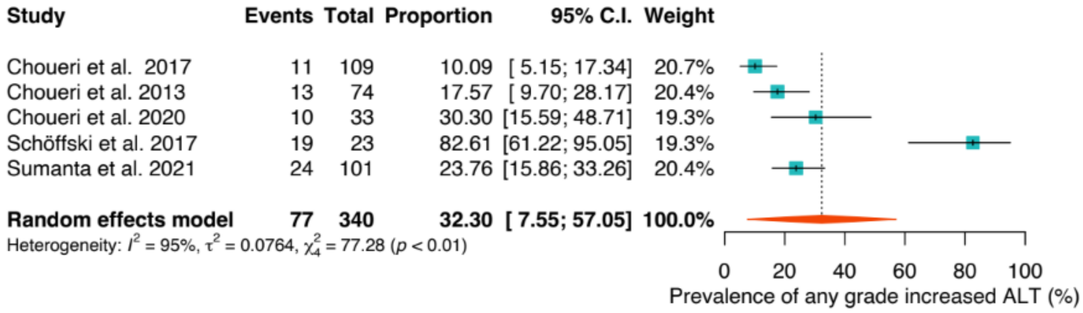

Figure S12. Grade ≥3 adverse events. **A.** Decreased WBC. **B.** Vomiting. **C.** Rash. **D.** Proteinuria. **E.** Decreased neutrophil Count. **F.** Nausea. **G.** Mucositis. **H.** Hyponatremia. **I.** Hypoalbuminemia. **J.** Hypertension. **K.** Fatigue. **L.** Extremity pain. **M.** Edema **N.** Dyspnea. **O.** Diarrhea. **P.** Increased creatinine. **Q.** Increased AST. **R.** Anorexia. **S.** Anemia. **T.** Increased ALT. **U.** Abdominal pain. Datas were sourced from reference [2,4–6].

A. Decreased WBC

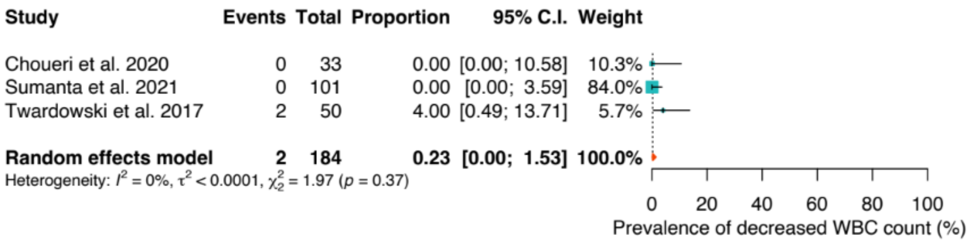

B. Vomiting

Study	Events	Total	Proportion	95% C.I.	Weight
Choueri et al. 2017	1	109	0.92	[0.02; 5.01]	25.1%
Choueri et al. 2013	0	74	0.00	[0.00; 4.86]	23.7%
Choueri et al. 2020	0	33	0.00	[0.00; 10.58]	4.9%
Schöffski et al. 2017	0	23	0.00	[0.00; 14.82]	2.5%
Sumanta et al. 2021	0	101	0.00	[0.00; 3.59]	43.8%
**Random effects model**	**1**	**340**	**0.23**	**[0.00; 1.13]**	**100.0%**

Heterogeneity: $I^2 = 0\%$, $\tau^2 = 0$, $\chi^2_4 = 0.76$ ($p = 0.94$)

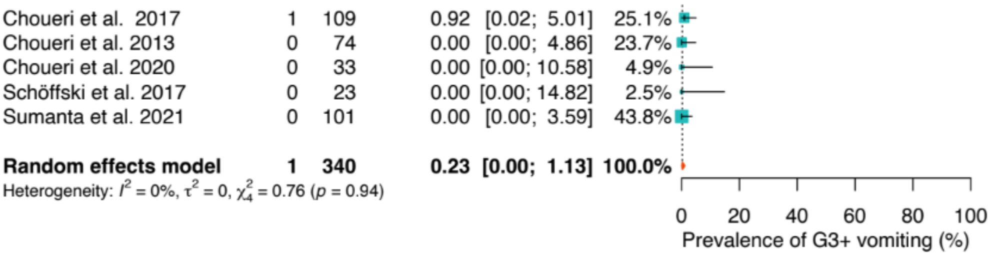

C. Rash

Study	Events	Total	Proportion	95% C.I.	Weight
Choueri et al. 2017	1	109	0.92	[0.02; 5.01]	59.1%
Schöffski et al. 2017	1	23	4.35	[0.11; 21.95]	2.7%
Sumanta et al. 2021	2	101	1.98	[0.24; 6.97]	25.6%
Twardowski et al. 2017	1	50	2.00	[0.05; 10.65]	12.6%
**Random effects model**	**5**	**283**	**1.42**	**[0.04; 2.80]**	**100.0%**

Heterogeneity: $I^2 = 0\%$, $\tau^2 = 0$, $\chi^2_3 = 1.03$ ($p = 0.79$)

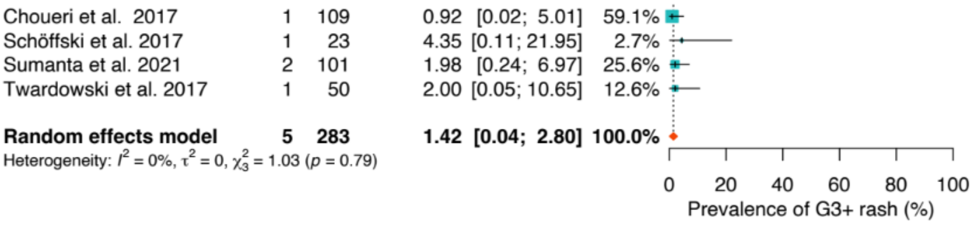

D. Proteinuria

Study	Events	Total	Proportion	95% C.I.	Weight
Choueri et al. 2017	1	109	0.92	[0.02; 5.01]	50.4%
Choueri et al. 2013	4	74	5.41	[1.49; 13.27]	6.2%
Sumanta et al. 2021	1	101	0.99	[0.03; 5.39]	43.4%
**Random effects model**	**6**	**284**	**1.23**	**[0.00; 2.51]**	**100.0%**

Heterogeneity: $I^2 = 26\%$, $\tau^2 < 0.0001$, $\chi^2_2 = 2.70$ ($p = 0.26$)

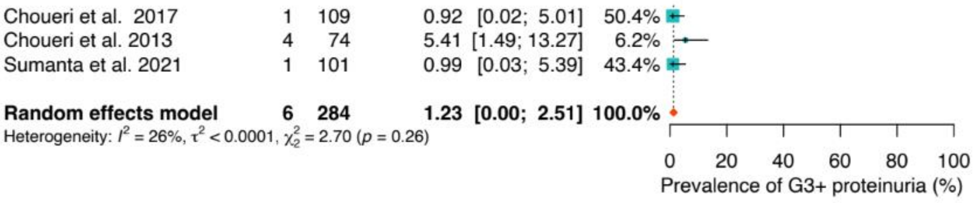

E. Decreased neutrophil count

Study	Events	Total	Proportion	95% C.I.	Weight
Choueri et al. 2020	0	33	0.00	[0.00; 10.58]	8.7%
Schöffski et al. 2017	0	23	0.00	[0.00; 14.82]	4.4%
Sumanta et al. 2021	0	101	0.00	[0.00; 3.59]	77.5%
Twardowski et al. 2017	1	50	2.00	[0.05; 10.65]	9.5%
**Random effects model**	**1**	**207**	**0.19**	**[0.00; 1.38]**	**100.0%**

Heterogeneity: $I^2 = 0\%$, $\tau^2 = 0$, $\chi^2_3 = 0.92$ ($p = 0.82$)

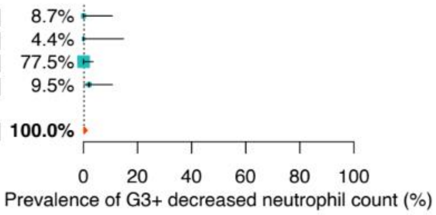

F. Nausea

Study	Events	Total	Proportion	95% C.I.	Weight
Choueri et al. 2017	0	109	0.00	[0.00; 3.33]	42.8%
Choueri et al. 2013	1	74	1.35	[0.03; 7.30]	9.8%
Choueri et al. 2020	0	33	0.00	[0.00; 10.58]	4.1%
Schöffski et al. 2017	0	23	0.00	[0.00; 14.82]	2.1%
Sumanta et al. 2021	0	101	0.00	[0.00; 3.59]	36.8%
Twardowski et al. 2017	1	50	2.00	[0.05; 10.65]	4.5%
**Random effects model**	**2**	**390**	**0.22**	**[0.00; 1.04]**	**100.0%**

Heterogeneity: $I^2 = 0\%$, $\tau^2 = 0$, $\chi^2_5 = 1.75$ ($p = 0.88$)

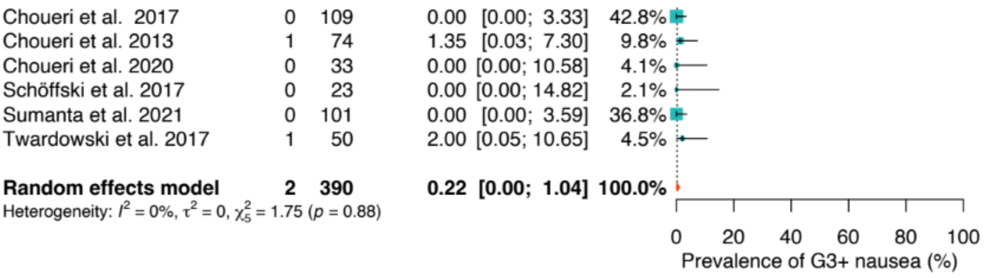

G. Mucositis

Study	Events	Total	Proportion	95% C.I.	Weight
Choueri et al. 2017	0	109	0.00	[0.00; 3.33]	65.8%
Choueri et al. 2020	0	33	0.00	[0.00; 10.58]	6.3%
Sumanta et al. 2021	1	101	0.99	[0.03; 5.39]	27.9%
**Random effects model**	**1**	**243**	**0.28**	**[0.00; 1.30]**	**100.0%**

Heterogeneity: $I^2 = 0\%$, $\tau^2 = 0$, $\chi^2_2 = 0.73$ ($p = 0.69$)

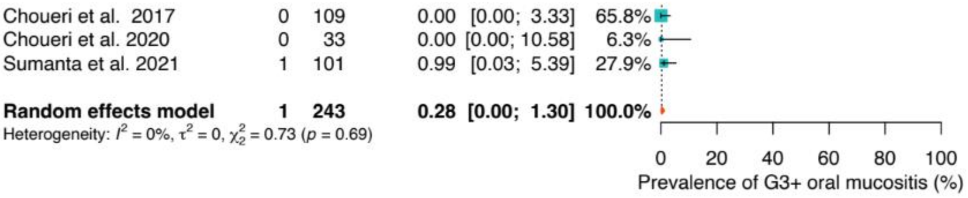

H. Hyponatremia

Study	Events	Total	Proportion	95% C.I.	Weight
Choueri et al. 2017	3	109	2.75	[0.57; 7.83]	60.5%
Schöffski et al. 2017	2	23	8.70	[1.07; 28.04]	6.3%
Sumanta et al. 2021	6	101	5.94	[2.21; 12.48]	33.2%
**Random effects model**	**11**	**233**	**4.18**	**[1.24; 7.13]**	**100.0%**

Heterogeneity: $I^2 = 0\%$, $\tau^2 = 0.0001$, $\chi^2_2 = 1.95$ ($p = 0.38$)

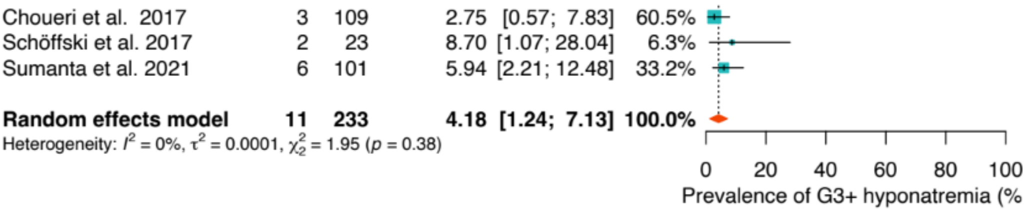

I. Hypoalbuminemia

Study	Events	Total	Proportion	95% C.I.	Weight
Choueri et al. 2017	0	109	0.00	[0.00; 3.33]	67.9%
Schöffski et al. 2017	0	23	0.00	[0.00; 14.82]	3.3%
Sumanta et al. 2021	1	101	0.99	[0.03; 5.39]	28.8%
**Random effects model**	**1**	**233**	**0.29**	**[0.00; 1.32]**	**100.0%**

Heterogeneity: $I^2 = 0\%$, $\tau^2 = 0$, $\chi^2_2 = 0.72$ ($p = 0.70$)

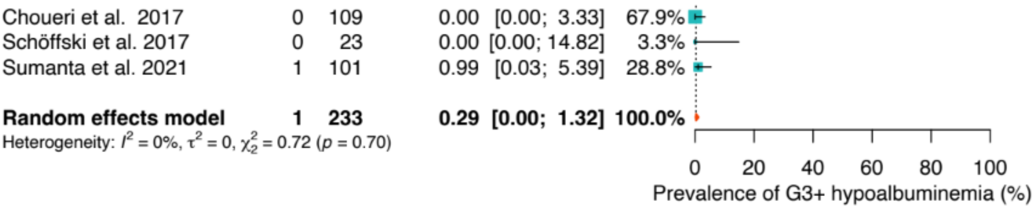

J. Hypertension

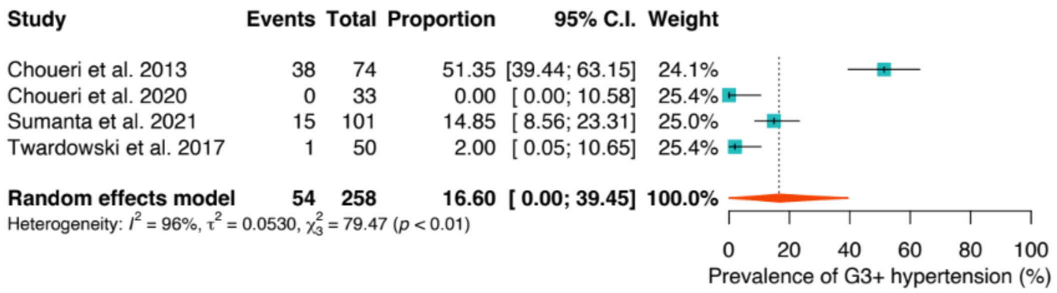

K. Fatigue

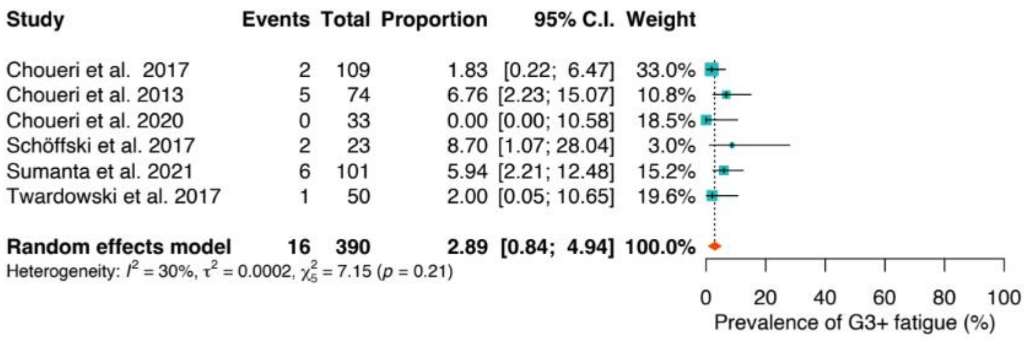

L. Extremity pain

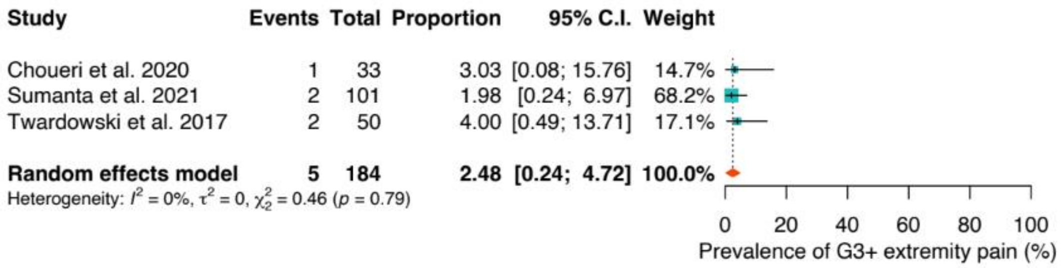

M. Edema

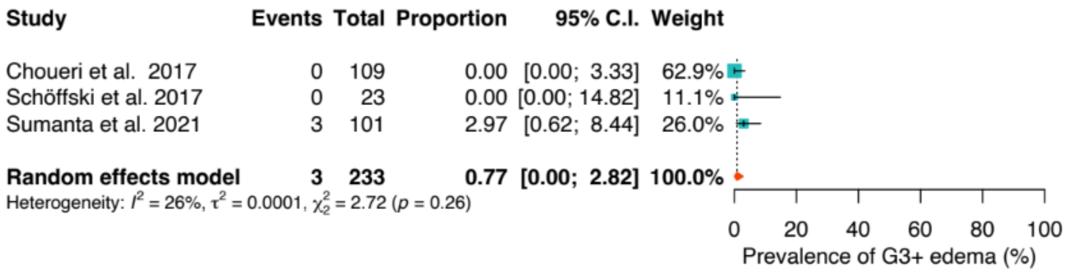

N. Dyspnea

Study	Events	Total	Proportion	95% C.I.	Weight
Choueri et al. 2020	1	33	3.03	[0.08; 15.76]	13.0%
Schöffski et al. 2017	2	23	8.70	[1.07; 28.04]	3.6%
Sumanta et al. 2021	1	101	0.99	[0.03; 5.39]	68.6%
Twardowski et al. 2017	2	50	4.00	[0.49; 13.71]	14.8%
**Random effects model**	**6**	**207**	**1.98**	**[0.00; 4.19]**	**100.0%**

Heterogeneity: $I^2 = 0\%$, $\tau^2 < 0.0001$, $\chi^2_3 = 2.82$ ($p = 0.42$)

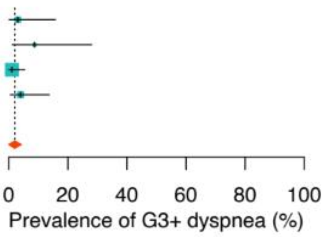

O. Diarrhea

Study	Events	Total	Proportion	95% C.I.	Weight
Choueri et al. 2017	0	109	0.00	[0.00; 3.33]	42.1%
Choueri et al. 2013	7	74	9.46	[3.89; 18.52]	7.2%
Choueri et al. 2020	0	33	0.00	[0.00; 10.58]	15.8%
Schöffski et al. 2017	0	23	0.00	[0.00; 14.82]	9.4%
Sumanta et al. 2021	2	101	1.98	[0.24; 6.97]	25.5%
**Random effects model**	**9**	**340**	**1.19**	**[0.00; 3.12]**	**100.0%**

Heterogeneity: $I^2 = 54\%$, $\tau^2 = 0.0002$, $\chi^2_4 = 8.76$ ($p = 0.07$)

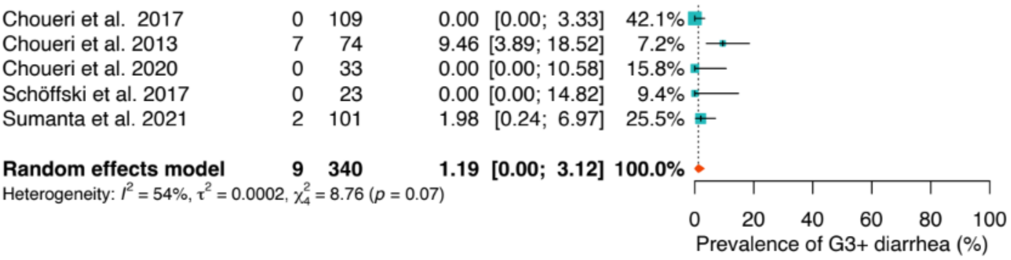

P. Increased creatinine

Study	Events	Total	Proportion	95% C.I.	Weight
Choueri et al. 2017	0	109	0.00	[0.00; 3.33]	78.3%
Choueri et al. 2013	1	74	1.35	[0.03; 7.30]	17.9%
Schöffski et al. 2017	0	23	0.00	[0.00; 14.82]	3.8%
**Random effects model**	**1**	**206**	**0.24**	**[0.00; 1.35]**	**100.0%**

Heterogeneity: $I^2 = 0\%$, $\tau^2 = 0$, $\chi^2_2 = 0.83$ ($p = 0.66$)

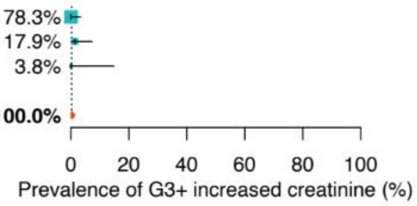

Q. Increased AST

Study	Events	Total	Proportion	95% C.I.	Weight
Choueri et al. 2017	3	109	2.75	[0.57; 7.83]	20.1%
Choueri et al. 2013	2	74	2.70	[0.33; 9.42]	13.9%
Choueri et al. 2020	4	33	12.12	[3.40; 28.20]	1.5%
Schöffski et al. 2017	3	23	13.04	[2.78; 33.59]	1.0%
Sumanta et al. 2021	1	101	0.99	[0.03; 5.39]	50.9%
Twardowski et al. 2017	1	50	2.00	[0.05; 10.65]	12.6%
**Random effects model**	**14**	**390**	**2.00**	**[0.62; 3.38]**	**100.0%**

Heterogeneity: $I^2 = 29\%$, $\tau^2 < 0.0001$, $\chi^2_5 = 7.07$ ($p = 0.22$)

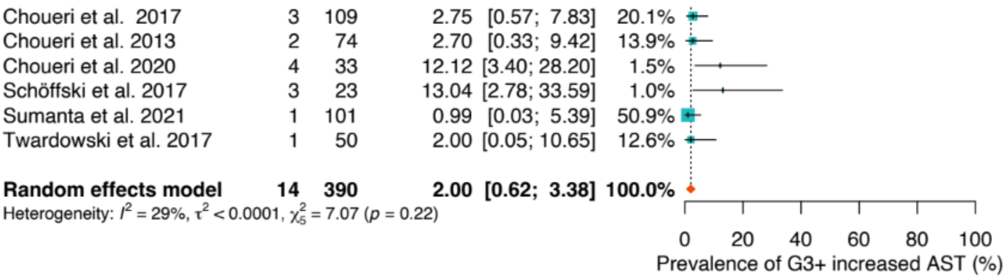

R. Anorexia

Study	Events	Total	Proportion	95% C.I.	Weight
Choueri et al. 2017	1	109	0.92	[0.02; 5.01]	39.0%
Choueri et al. 2013	1	74	1.35	[0.03; 7.30]	18.1%
Choueri et al. 2020	0	33	0.00	[0.00; 10.58]	7.6%
Schöffski et al. 2017	1	23	4.35	[0.11; 21.95]	1.8%
Sumanta et al. 2021	1	101	0.99	[0.03; 5.39]	33.5%
**Random effects model**	**4**	**340**	**1.01**	**[0.00; 2.13]**	**100.0%**

Heterogeneity: $I^2 = 0\%$, $\tau^2 = 0$, $\chi^2_4 = 0.93$ ($p = 0.92$)

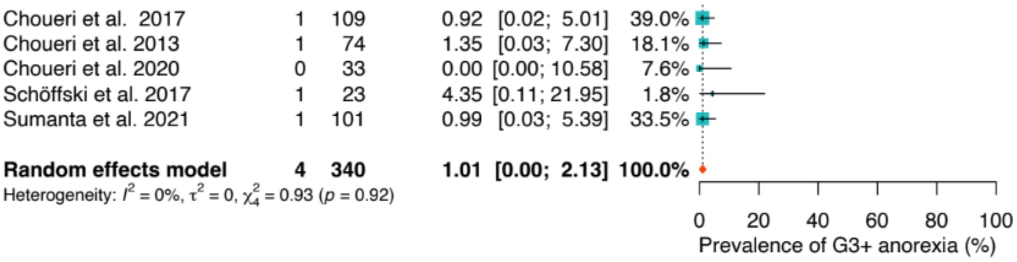

S. Anemia

Study	Events	Total	Proportion	95% C.I.	Weight
Choueri et al. 2017	1	109	0.92	[0.02; 5.01]	44.2%
Choueri et al. 2020	0	33	0.00	[0.00; 10.58]	8.7%
Schöffski et al. 2017	0	23	0.00	[0.00; 14.82]	4.3%
Sumanta et al. 2021	1	101	0.99	[0.03; 5.39]	38.0%
Twardowski et al. 2017	2	50	4.00	[0.49; 13.71]	4.8%
**Random effects model**	**4**	**316**	**0.97**	**[0.00; 2.16]**	**100.0%**

Heterogeneity: $I^2 = 0\%$, $\tau^2 = 0$, $\chi^2_4 = 1.53$ ($p = 0.82$)

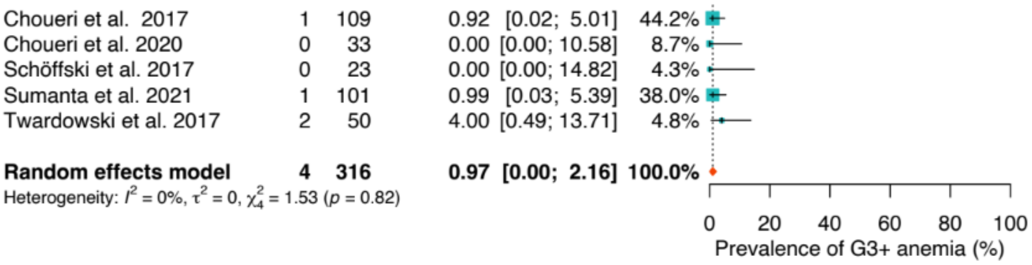

T. Increased ALT

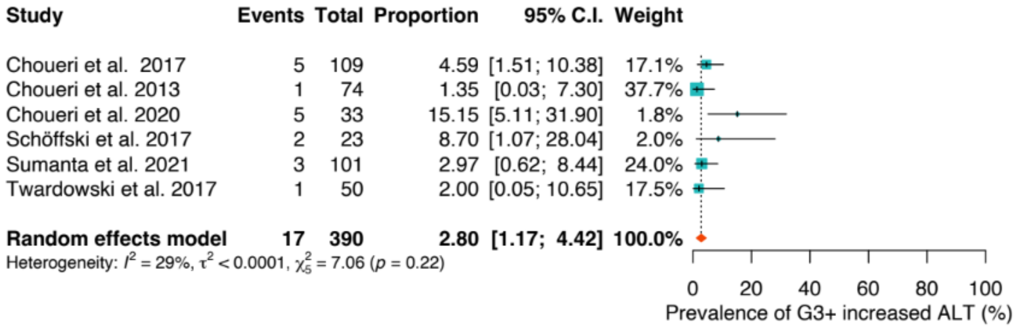

U. Abdominal pain

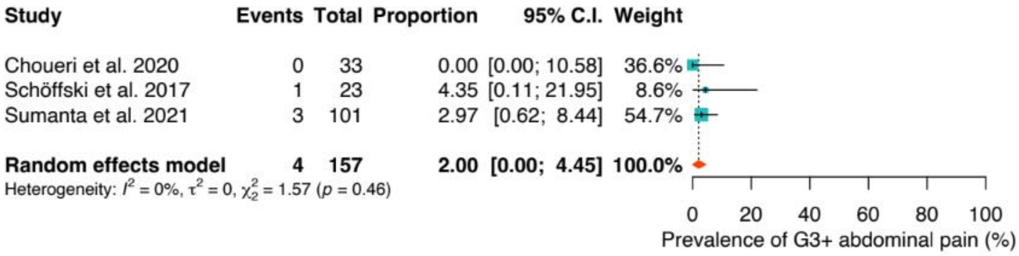

Reference

1. Twardowski, P.W.; Tangen, C.M.; Wu, X.; Plets, M.R.; Plimack, E.R.; Agarwal, N.; Vogelzang, N.J.; Wang, J.; Tao, S.; Thompson, I.M.; et al. Parallel (Randomized) Phase II Evaluation of Tivantinib (ARQ197) and Tivantinib in Combination with Erlotinib in Papillary Renal Cell Carcinoma: SWOG S1107. *Kidney Cancer* **2017**, *1*, 123–132. https://doi.org/10.3233/KCA-170018.
2. Pal, S.K.; Tangen, C.; Thompson, I.M.; Balzer-Haas, N.; George, D.J.; Heng, D.Y.C.; Shuch, B.; Stein, M.; Tretiakova, M.; Humphrey, P.; et al. A Comparison of Sunitinib with Cabozantinib, Crizotinib, and Savolitinib for Treatment of Advanced Papillary Renal Cell Carcinoma: A Randomised, Open-Label, Phase 2 Trial. *Lancet* **2021**, *397*, 695–703. https://doi.org/10.1016/S0140-6736(21)00152-5.
3. Choueiri, T.K.; Heng, D.Y.C.; Lee, J.L.; Cancel, M.; Verheijen, R.B.; Mellemgaard, A.; Ottesen, L.H.; Frigault, M.M.; L'Hernault, A.; Szijgyarto, Z.; et al. Efficacy of Savolitinib vs Sunitinib in Patients With MET-Driven Papillary Renal Cell Carcinoma: The SAVOIR Phase 3 Randomized Clinical Trial. *JAMA Oncol.* **2020**, *6*, 1247–1255. https://doi.org/10.1001/jamaoncol.2020.2218.
4. Schöffski, P.; Wozniak, A.; Escudier, B.; Rutkowski, P.; Anthoney, A.; Bauer, S.; Sufliarsky, J.; van Herpen, C.; Lindner, L.H.; Grünwald, V.; et al. Crizotinib Achieves Long-Lasting Disease Control in Advanced Papillary Renal-Cell Carcinoma Type 1 Patients with MET Mutations or Amplification. EORTC 90101 CREATE Trial. *Eur. J. Cancer* **2017**, *87*, 147–163. https://doi.org/10.1016/j.ejca.2017.10.014.
5. Choueiri, T.K.; Vaishampayan, U.; Rosenberg, J.E.; Logan, T.F.; Harzstark, A.L.; Bukowski, R.M.; Rini, B.I.; Srinivas, S.; Stein, M.N.; Adams, L.M.; et al. Phase II and Biomarker Study of the Dual MET/VEGFR2 Inhibitor Foretinib in Patients with Papillary Renal Cell Carcinoma. *J. Clin. Oncol.* **2013**, *31*, 181–186. https://doi.org/10.1200/JCO.2012.43.3383.
6. Choueiri, T.K.; Plimack, E.; Arkenau, H.-T.; Jonasch, E.; Heng, D.Y.C.; Powles, T.; Frigault, M.M.; Clark, E.A.; Handzel, A.A.; Gardner, H.; et al. Biomarker-Based Phase II Trial of Savolitinib in Patients With Advanced Papillary Renal Cell Cancer. *J. Clin. Oncol.* **2017**, *35*, 2993–3001. https://doi.org/10.1200/JCO.2017.72.2967.
7. Martínez Chanzá, N.; Xie, W.; Asim Bilen, M.; Dzimitrowicz, H.; Burkart, J.; Geynisman, D.M.; Balakrishnan, A.; Bowman, I.A.; Jain, R.; Stadler, W.; et al. Cabozantinib in Advanced Non-Clear-Cell Renal Cell Carcinoma: A Multicentre, Retrospective, Cohort Study. *Lancet Oncol.* **2019**, *20*, 581–590. https://doi.org/10.1016/S1470-2045(18)30907-0.
8. Tachibana, H.; Inakawa, T.; Nemoto, Y.; Ishihara, H.; Fukuda, H.; Yoshida, K.; Iizuka, J.; Hashimoto, Y.; Tanabe, K.; Kondo, T.; et al. Efficacy of Cabozantinib for Papillary Compared with Clear-Cell Renal Cell Carcinoma Following Immune Checkpoint Inhibitor Treatment. *Anticancer Res.* **2022**, *42*, 3151–3158. https://doi.org/10.21873/anticanres.15804.
9. Choueiri, T.K.; Heng, D.Y.C.; Lee, J.L.; Cancel, M.; Verheijen, R.B.; Mellemgaard, A.; Ottesen, L.H.; Frigault, M.M.; L'Hernault, A.; Szijgyarto, Z.; et al. Efficacy of Savolitinib vs Sunitinib in Patients With MET-Driven Papillary Renal Cell Carcinoma: The SAVOIR Phase 3 Randomized Clinical Trial. *JAMA Oncol.* **2020**, *6*, 1247–1255. https://doi.org/10.1001/jamaoncol.2020.2218.
10. Suárez, C.; Larkin, J.M.G.; Patel, P.; Valderrama, B.P.; Rodriguez-Vida, A.; Glen, H.; Thistlethwaite, F.; Ralph, C.; Srinivasan, G.; Mendez-Vidal, M.J.; et al. Phase II Study Investigating the Safety and Efficacy of Savolitinib and Durvalumab in Metastatic Papillary Renal Cancer (CALYPSO). *J. Clin. Oncol.* **2023**, *41*, 2493–2502. https://doi.org/10.1200/JCO.22.01414.
